# Is It Necessary to Use Cement Augmentation in Internal Fixation for Trochanteric Fractures in the Elderly? A Systematic Review and Meta-Analysis

**DOI:** 10.1055/s-0046-1824532

**Published:** 2026-08-14

**Authors:** Adhe Sugandhi, Ni Kadek Saras Dwi Guna, Grace Yulia Alphani Yapson, I Putu Divanaya Suryanov, I Gusti Ngurah Paramartha Wijaya Putra

**Affiliations:** 1 General Practitioner, Universitas Udayana, Denpasar, Bali, Indonesia; 2 Department of Orthopedics and Traumatology, Ngoerah Hospital, Bali, Indonesia

**Keywords:** bone cements, elderly, fracture fixation, internal, hip fractures, cimentos ósseos, fixação interna de fraturas, fraturas do quadril, idoso

## Abstract

**Objective:**

To evaluate the efficacy of cement augmentation in internal fixation of trochanteric fractures in the elderly.

**Methods:**

A comprehensive search of the MEDLINE, SpringerLink, CENTRAL, and Scopus databases was conducted up to February 2025 for randomized clinical trials (RCTs) comparing internal fixation with and without cement augmentation. The review adhered to the 2020 Preferred Reporting Items for Systematic Reviews and Meta-Analyses (PRISMA) statement guidelines. Random-effects models were used to pool effect estimates. The primary outcomes were fixation failure and tip-apex distance (TAD). The secondary outcomes included mortality, reoperation rate, length of hospital stay, blood loss, and operative time. The risk of bias was assessed with the revised version of the Cochrane Risk of Bias tool (RoB 2.0; Cochrane Collaboration), and evidence certainty, with the Grading of Recommendations, Assessment, Development, and Evaluation (GRADE) approach.

**Results:**

Five RCTs comprising 509 patients were included. Cement augmentation did not significantly reduce fixation failure (relative risk [RR] = 1.13; 95%CI: 0.20–6.43) but significantly improved the TAD (mean difference [MD] = -2.11 mm; 95%CI: -3.13 to -1.09; *p* < 0.0001). There were no significant effects on mortality (RR = 1.10; 95%CI: 0.54–2.22) or reoperation (RR = 0.12; 95%CI: 0.01–2.09). Cement augmentation shortened the hospital stay by 2.5 days (MD = -2.59; 95%CI: -6.87 to 1.69), but it increased blood loss (MD = 49.01; 95%CI: -61.78 to 159.8) and operative time by 11 minutes (MD = 11.8; 95%CI: 5.91–17.69).

**Conclusions:**

Cement augmentation improves fixation quality and reduces hospitalization in elderly patients with trochanteric fractures, but it increases blood loss and operative time. Its use should be individualized based on patient risk profiles.

## Resumo

**Objetivo:**

Avaliar a eficácia do aumento com cimento na fixação interna de fraturas trocantéricas em idosos.

**Métodos:**

Uma busca abrangente nas bases de dados MEDLINE, SpringerLink, CENTRAL e Scopus sobre ensaios clínicos randomizados (ECRs) que comparavam a fixação interna com e sem aumento com cimento foi conduzida até fevereiro de 2025. A revisão seguiu as diretrizes da declaração Preferred Reporting Items for Systematic Reviews and Meta-Analyses (PRISMA) de 2020. Modelos de efeitos aleatórios foram utilizados para a combinação das estimativas de efeito. Os desfechos primários foram a falha de fixação e a distância ponta-ápice (DPA). Os desfechos secundários incluíram mortalidade, taxa de reoperação, tempo de internação, perda sanguínea e tempo operatório. O risco de viés foi avaliado com a versão revisada da ferramenta Cochrane Risk of Bias (RoB 2.0; Cochrane Collaboration), e a certeza da evidência foi analisada pelo método Grading of Recommendations, Assessment, Development, and Evaluation (GRADE).

**Resultados:**

Cinco ECRs foram incluídos, num total de 509 pacientes. O aumento com cimento não reduziu significativamente a falha de fixação (risco relativo [RR] = 1,13; IC95% = 0,20–6,43), mas melhorou significativamente a DPA (diferença média [DM] = -2,11 mm; IC95% = -3,13 a -1,09; *p* < 0,0001). Não houve efeito significativo sobre a mortalidade (RR = 1,10; IC95% = 0,54–2,22), nem sobre a taxa de reoperação (RR = 0,12; IC95% = 0,01–2,09). O aumento com cimento reduziu o tempo de internação em 2,5 dias (DM = -2,59; IC95% = -6,87 a 1,69), mas aumentou a perda sanguínea (DM = 49,01; IC95% = -61,78 a 159,8) e o tempo operatório em 11 minutos (DM = 11,8; IC95% = 5,91–17,69).

**Conclusão:**

O aumento com cimento melhora a qualidade da fixação e reduz o tempo de internação em pacientes idosos com fraturas trocantéricas, mas aumenta a perda sanguínea e o tempo operatório. Seu uso deve ser individualizado com base no perfil de risco do paciente.

## Introduction

Trochanteric fractures are a common orthopedic challenge in the elderly, primarily due to age-related bone fragility and osteoporosis. These fractures often require internal fixation; however, conventional fixation methods may be insufficient in severely-osteoporotic bone, leading to complications such as fixation failure, implant migration, and other complications. 1 2

Some internal fixation treatments for trochanteric fractures include the Gamma3 Nail (Stryker Corporation), Trochanteric Fixation Nail–Advanced (TFNA; DePuy Synthes Inc.), Proximal Femoral Nail Antirotation (PFNA; DePuy Synthes Inc.), Intertrochanteric Antegrade Nail (INTERTAN; Smith & Nephew plc), and Sliding/Dynamic Hip Screw (SHS/DHS; DePuy Synthes Inc.). 3 Each type of internal fixation has its own advantages and disadvantages. Fixation failure in DHS can reach up to 8.4%, while in cephalomedullary nailing it can be as high as 6.3%. 4 5 Therefore, additional techniques are needed to address this issue, one of which is the use of cement augmentation, either with polymethylmethacrylate (PMMA) or calcium phosphate cement (CPC). 6 7 However, some studies suggest that cement augmentation is necessary, while others indicate that its addition is not significant and may even increase the rate of complications, such as bleeding. 8 9 10 11 12 Based on this, further studies are needed to draw a definitive conclusion on this issue.

The present study aims to determine whether cement augmentation is necessary in internal fixation for trochanteric fractures in the elderly by evaluating its impact on fixation failure, the need for revision surgery, and other complications.

## Methods

The current meta-analysis followed the requirements of the *Cochrane Handbook for Systematic Review of Interventions* , and it was registered in the International Prospective Register of Systematic Reviews (PROSPERO; CRD420251006740). As the review did not constitute a clinical trial, ethical approval was not deemed necessary.

The present study included randomized clinical trials (RCT) on cement augmentation of internal fixation for trochanteric fractures in the elderly. The inclusion criteria were patients older than 65 years with trochanteric fractures, internal fixation with the TFNA, PFNA, Gamma3 nail, or SHS/DHS, and a minimum of 6 months of follow-up. And the exclusion criteria were cadaveric studies, reviews, meta-analyses, and case reports, pathologic, open, periprosthetic, or non-trochanteric fractures, and unknown follow-up, non-traumatic fractures, non-union, or revision surgeries.

### Literature Search Strategy

The current study was written according to the guidelines of the 2020 Preferred Reporting Items for Systematic Review and Meta-Analyses (PRISMA) statement. Two independent clinical researchers (AS and NKSDG) searched online databases such as MEDLINE, SpringerLink, CENTRAL, and Scopus on February 24th, 2025 **.** To minimize the risk of missing relevant research, no filters were applied to the search strategy. The detailed search strategy is provided in the Supplementary Material.

### Data Extraction and Risk of Bias Assessment

Two authors (AS and NKSDG) extracted and recorded relevant data from each included study following a standardized protocol. The extracted information included the authors and year of publication, country, study period, device, type of cement, patient demographics (age, gender, fracture type), and follow-up duration. Additionally, the incidence of intraoperative complications and the need for further surgery were calculated for each study.

Two authors independently assessed the risk of bias in the included studies using the revised Cochrane Risk of Bias tool (RoB 2.0; Cochrane Collaboration). Any discrepancies were resolved through discussion, or, if necessary, consultation with a third evaluator. The RoB 2.0 evaluates five domains: bias arising from the randomization process, deviations from intended interventions, missing outcome data, measurement of outcomes, and selective reporting. Each domain is rated as presenting *low risk* , *some concerns* , or *high risk* of bias. An overall risk of bias assessment was determined separately for each outcome in each study, based on evaluations across all domains.

### Outcome Measures

The primary outcomes were fixation failure and tip-apex distance (TAD) measurement, while the secondary outcomes included mortality, reoperation, hospital stay, blood loss, and operative time.

### Data Analysis

The statistical analysis was conducted on the integrated development environment RStudio (free and open source) for the development of a meta-analysis with an evaluation of dichotomous and continuous variables. For the dichotomous outcomes—fixation failure, mortality, and reoperation—we calculated the relative risk (RR) with 95%CIs. For the continuous variables—TAD, length of hospital stay, blood loss, and operative time—we calculated the mean difference (MD) with 95%CIs.

Statistical heterogeneity was estimated using the I ^2^ statistic and between-study variance (Tau ^2^ ), with additional assessment using the Cochran's Q test. Heterogeneity was further evaluated by visual inspection of forest plots, and the I ^2^ statistic was interpreted as low (0–40%), moderate (30–60%), substantial (50–90%), and considerable (75–100%). When I ^2^ exceeded 50%, we investigated potential sources of heterogeneity. The Cochrane's Q test was used to assess heterogeneity, with statistical significance set at *p* < 0.10.

We applied a random-effects model for all analyses, as it accounts for variability across studies. Additionally, we conducted sensitivity analyses to explore potential heterogeneity and assess the impact of bias on effect estimates. Statistical significance for the main analyses was set at *p* < 0.05.

## Results

### Search Results

**Fig. 1:**
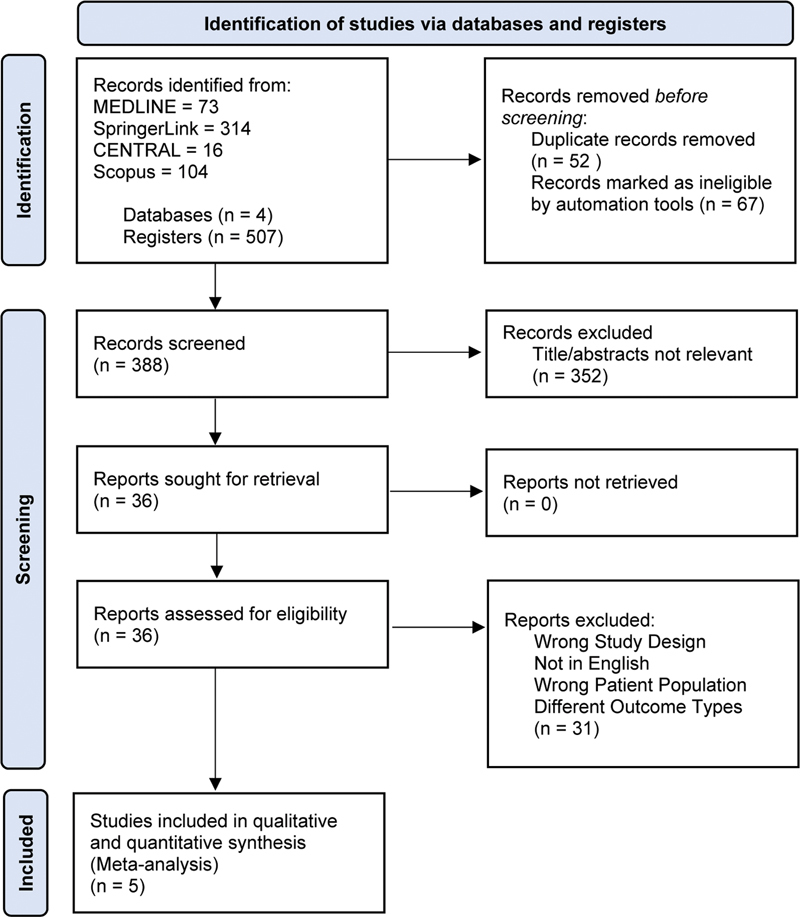
Flow diagram of the literature search results according to the according to the 2020 PRISMA Statement Preferred Reporting Items for Systematic Reviews and Meta-Analyses (PRISMA) statement.

A total of 512 references were identified and assessed. A comprehensive overview of the search and selection process is illustrated in Fig. 1 . After removal of duplicate records and records deemed ineligible by automation tools, 388 records were screened by title and abstract, 352 of which were excluded as not relevant. A total of 36 full-text articles were assessed for eligibility, and 31 were excluded due to inappropriate study design, language other than English, incorrect patient population, or different outcome measures. Ultimately, five randomized controlled trials 8 9 10 11 12 met the inclusion criteria and were included in the qualitative and quantitative synthesis (meta-analysis).

### Study Characteristics

We searched 4 databases and included 5 studies 8 9 10 11 12 (1997–2019), from Sweden, Italy, Germany, China, and Colombia, on cement augmentation in proximal femoral fracture fixation using the DHS, Gamma3, PFNA, TFNA, and the Aimstrong Proximal Femoral Nail (APFN, Double Medical Technology Inc.). As summarized in Table 1 , PMMA was used in 3 studies, 8 10 11 CPC, in one, 12 and, in the remaining study, 9 the material was not reported. Cement volumes ranged from 2 to 20 ml; 2 studies 9 10 did not report the amounts.

**Table 1:** Characteristics of the studies included

Author (year)	Country	Study period	Device	Type of cement	Cement injected	Total sample size		Gender (F/M)		Mean age (years)		AO31(A1/A2/A3)-AO32A2		Follow-up
						aug	control	aug	control	aug	control	aug	control	
Mattsson et al. 12 (2005)	Sweden	1997–2000	DHS	Calcium Phosphate	20 mL	55	57	44/11	47/10	81.2 ± 7.0	82.0 ± 6.3	NR	NR	6 months
Dall'Oca et al. 11 (2010)	Italy	January 2006–March 2010	Gamma3 nail	PMMA	2–3 mL	40	40	26/14	30/10	85.3 ± 2.3	82.3 ± 1.2	0/20/15	0/22/14	12 months
Kammerlander et al. 10 (2018)	Germany	March 2012–July 2015	PFNA	PMMA	NR	87	135	99/19	86/21	86.1 ± 4.6	85.6 (4.9)	0/9/9	0/96/22	12 months
Ni et al. 9 (2022)	China	January 2017–January 2019	PFNA and APFN	NR	NR	22	20	NR	NR	NR	NR	NR	NR	12–16 months
Salazar et al. 8 (2023)	Colombia	September 2015–December 2017	TFNA	PMMA	4 mL	23	30	19/4	23/7	83.87	85.91	5/15/2/1	8/7/12/3	12 months

**Abbreviations:** APFN, Aimstrong Proximal Femoral Nail; aug, augmentation; DHS, Dynamic Hip Screw; F, female; M, male; NR, not reported; PFNA, Proximal Femoral Nail Antirotation; PMMA, polymethylmethacrylate; TFNA, Trochanteric Fixation Nail–Advanced.

The augmented groups had 22 to 87 patients; the control groups had 20 to 135 subjects. Most participants were elderly female individuals (mean age: 81.2–86.1 years in the augmented groups, and 82.0–85.9 among the controls). Three studies 8 9 10 reported the Arbeitsgemeinschaft für Osteosynthesefragen (AO) classifications (mainly AO31-A2/A3). Follow-up generally lasted 12 months; in 1 study, 12 it lasted 6 months, and, in another, 9 12 to 16 months.

### Risk of Bias Assessment

**Fig. 2:**
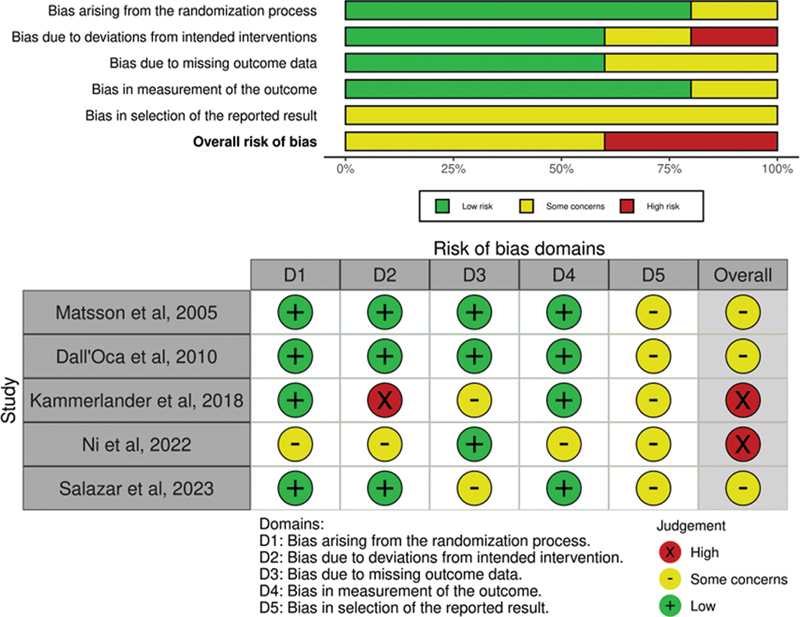
Risk-of-bias regarding the outcome.

Two authors (AS and IPDS) independently assessed the risk of bias for the outcomes of fixation failure and TAD using the RoB 2.0 tool ( Fig. 2 ). Three studies presented low risk of bias or some concerns, while two 9 10 were deemed to have a high overall risk of bias, mainly related to deviations from intended interventions and outcome measurement. Bias arising from the randomization process and missing outcome data was generally low. As commonly observed in surgical trials, blinding-related domains represented the main sources of potential bias; however, the primary outcomes, fixation failure and TAD, are objective measures and are, therefore, less susceptible to performance and detection bias. The presence of high-risk studies and small sample sizes may have contributed to imprecision in several outcomes, and the overall certainty of evidence should be interpreted with caution.

### Meta-analysis Results

The comparison of the outcomes of cement augmentation in internal fixation for trochanteric fractures are summarized in Table 2 and in **Table S3 of the Supplementary Material** .

**Table 2:** Summary of Findings

Outcome	Anticipated absolute effects (95%CI)	Relative effect (95%CI)	No. of participants (studies)	Certainty of evidence (GRADE)	Comments
Fixation failure	2 more per 1,000 (from 14 fewer to 98 more)	RR 1.13 (0.20–6.43)	387 (3 RCTs)	Low ^a,b^	Very uncertain about the effect on fixation failure
Tip–apex distance	MD = 2.11 mm lower (3.13–1.09 lower)	–	245 (3 RCTs)	High ^a^	Likely reduction in TAD with cement augmentation
Mortality (1 year)	9 more per 1,000 (from 42 fewer to 112 more)	RR 1.10 (0.54–2.22)	302 (2 RCTs)	Low ^a,b^	Little to no difference in mortality
Reoperation	39 fewer per 1,000 (from 44 fewer to 48 fewer)	RR 0.12 (0.01–2.09)	222 (2 RCTs)	Low ^a^	Cement augmentation may reduce the reoperation rate
Length of hospital stay	MD = 2.59 days lower (6.87 lower to 1.69 lower)	–	234 (3 RCTs)	Low ^a,c^	Cement augmentation may slightly reduce the length of hospital stay
Blood loss	MD = 49.01 ml higher (61.78 lower to 159.8 higher)	–	154 (3 RCTs)	Low ^a,c^	Evidence uncertain for blood loss difference
Operative time	MD = 11.8 min higher (5.91–17.69 higher)	–	234 (3 RCTs)	Moderate ^a,c^	Cement augmentation increases the operative time

**Abbreviations:** GRADE, Grading of Recommendations, Assessment, Development, and Evaluation; MD, mean difference; min, minutes; RCT, randomized clinical trial; RR, relative risk; TAD, tip-apex distance.

**Notes:**^a^ Participants and personnel were not blinded; ^b^ low robustness; e ^c^ high heterogeneity.

#### Primary Outcomes

##### Fixation Failure

**Fig. 3:**
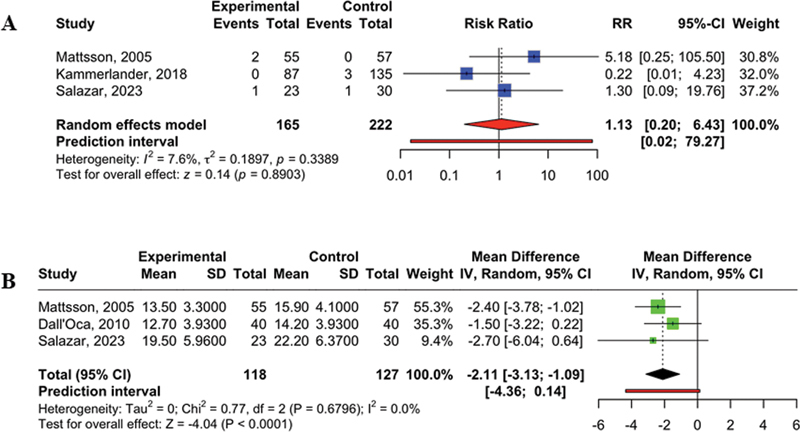
Forest plot of the comparison of primary outcomes: ( **A** ) Fixation failure; and ( **B** ) tip-apex distance.

Data from 3 studies 8 10 12 involving 387 participants were combined in this meta-analysis to assess fixation failures. In the cement augmentation group, one case of cut-through was reported, 8 along with two cases of plate loosening. 12 The pooled analysis showed no significant benefit of cement augmentation, with a wide confidence interval (RR = 1.13; 95%CI: 0.20–6.43; *p* = 0.8903) The certainty of evidence was rated as low. Additionally, there was no indication of substantial statistical heterogeneity (Tau ^2^ = 0.1897; I ^2^ = 7.6%) ( Fig. 3A ; **Supplementary Material, Fig. S1** ).

##### Tip Apex Distance

Three studies 8 11 12 (245 participants) reported data on TAD. The analysis showed that the cement augmentation group had a significantly lower TAD compared to the non-augmented group, with a mean difference of -2.11 mm (95%CI: -3.13 to -1.09; *p* < 0.0001). This indicates that cement augmentation may improve the accuracy of implant placement. There was no heterogeneity among the included studies (Tau ^2^ = 0; I ^2^ = 0%; *p* = 0.6796) ( Fig. 3B ; **Supplementary Material, Fig. S2** ).

#### Secondary Outcomes

##### Mortality

**Fig. 4:**
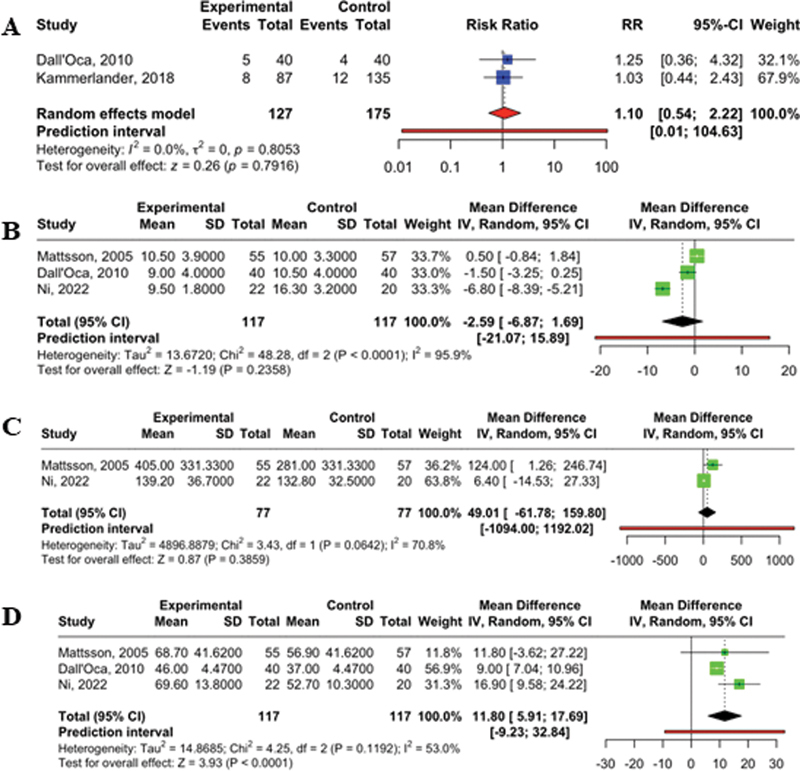
Forest plot of the comparison of secondary outcomes: ( **A** ) Mortality; ( **B** ) length of hospital stay; ( **C** ) blood loss; and ( **D** ) operative time.

Two studies 10 11 provided data on one-year mortality, which were combined for ease of analysis. The mortality rate in the cement augmentation group was 10.2%, compared to 9.1% in the non-augmented group. However, there was no statistically significant difference in postoperative mortality between the two groups (RR = 1.10; 95%CI: 0.54–2.22; *p* = 0.7916; I ^2^ = 0%) ( Fig. 4A ).

##### Reoperation

Reoperation was reported in two studies. 10 12 However, only one study 10 provided this outcome separately for both treatment groups, showing no significant difference between them (RR = 0.12; 95%CI: 0.01–2.09; *p* = 0.1454) ( Fig. 4B **)** and the evidence was considered of low quality. Heterogeneity was not applicable in this case.

##### Length of Hospital Stay

Data from 3 studies 9 11 12 (117 participants in each group) were included in the meta-analysis to compare the length of hospital stay between the cement augmentation group and the control group. The results showed an MD of -2.59 days in favor of the cement augmentation group; however, this difference was not statistically significant (MD = -2.59, 95%CI: -6.87 to 1.69; *p* = 0.2358). The prediction interval ranged widely, from -21.07 to 15.89, indicating considerable variability in the possible true effect across different settings. Substantial statistical heterogeneity was observed (Tau ^2^ = 13.6720; I ^2^ = 95.9%; Chi ^2^ = 48.28; degrees of freedom [df] = 2; *p* < 0.0001) ( Fig. 4B ).

##### Blood Loss

Two studies 9 12 (77 participants per group) reported intraoperative blood loss. The pooled results showed an MD of 49.01 mL in favor of the control group; however, this difference was not statistically significant (MD = 49.01, 95%CI: -61.78 to 159.80; *p* = 0.3859). The prediction interval was wide (-1094.00 to 1192.02), indicating a high degree of uncertainty regarding the true effect across different settings. Moderate to substantial heterogeneity was observed among the included studies (Tau ^2^ = 4896.8879; I ^2^ = 70.8%; Chi ^2^ = 3.43, df = 1; *p* = 0.0642) ( Fig. 4C ).

##### Operative Time

A total of 3 studies 9 11 12 (117 participants per group) reported on operative time. 9 11 12 The pooled analysis showed a statistically significant longer operative time in the cement augmentation group compared to the non-augmented group, with an MD of 11.80 minutes (95% CI: 5.91–17.69; *p* < 0.0001). The prediction interval ranged from -9.23 to 32.84, suggesting some variability in the true effect across settings. Moderate heterogeneity was detected (Tau ^2^ = 14.8685; I ^2^ = 53.0%), though it was not statistically significant (Chi ^2^ = 4.25; df = 2; *p* = 0.1192) ( Fig. 4D ).

### Publication Bias

Due to the small number of included studies 8 9 10 11 12 (n = 5), a formal assessment of publication bias using funnel-plot visualization or statistical tests such as the Egger's or Begg's tests was not performed. According to *Cochrane Handbook* recommendations, at least 10 studies are generally required to adequately detect asymmetry and assess publication bias. With fewer studies, funnel plots and regression-based methods lack sufficient power and can lead to misleading interpretations. Therefore, since the publication bias cannot be formally evaluated in the current analysis, it is a potential limitation of the present findings.

### Sensitivity Analysis

**Fig. 5:**
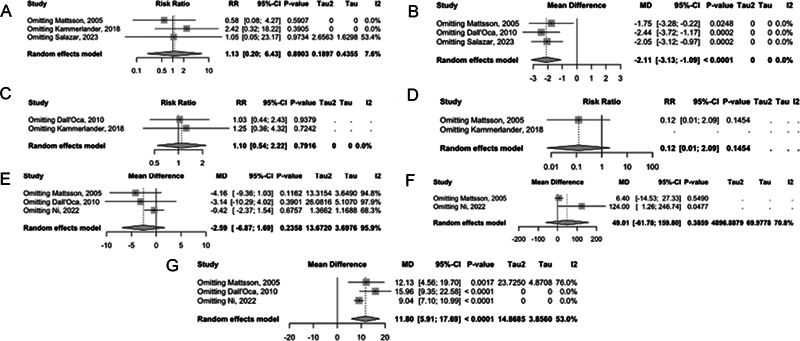
Leave-one-out analysis: ( **A** ) fixation failure; ( **B** ) tip-apex distance; ( **C** ) mortality; ( **D** ) reOperation; ( **E** ) length of hospital stay; ( **F** ) blood loss; and ( **G** ) operative time.

A sensitivity analysis using the leave-one-out approach was performed to evaluate the robustness of the pooled estimates across all outcomes ( Fig. 5 ). The analysis demonstrated that the significant reduction in TAD (MD = -2.11; 95%CI: -3.13 to -1.09; *p* < 0.0001) and the increase in operative time (MD = 11.80; 95%CI: 5.91–17.69; *p* < 0.0001) remained stable, indicating consistent findings regardless of which study was omitted. In contrast, outcomes such as fixation failure (RR = 1.13; 95%CI: 0.20–6.43; *p* = 0.89), mortality (RR = 1.10; 95%CI: 0.54–2.22; *p* = 0.79), reoperation (RR = 0.12; 95%CI: 0.01–2.09; *p* = 0.15), length of hospital stay (MD = -2.59; 95%CI: -6.87 to 1.69; *p* = 0.24), and blood loss (MD = 49.01; 95%CI: -61.78 to 159.80; *p* = 0.39) showed no statistically significant effects and varied in heterogeneity, with length of hospital stay and blood loss exhibiting particularly high between-study variance (I ^2^ = 95.9% and 70.8% respectively). These findings suggest that, while certain operative parameters such as TAD and operative time are reliably affected by the intervention, other outcomes may be influenced by study-specific factors or lack sufficient power to detect a meaningful difference.

## Discussion

The current meta-analysis found that cement augmentation in internal fixation for trochanteric fractures significantly improved implant positioning, as indicated by reduced TAD. However, there was no statistically significant benefit in reducing fixation failure, reoperation, or mortality. As a result, while the mechanical stabilization hypothesis is partially supported, the overall hypothesis that cement augmentation improves clinical outcomes is only partially accepted.

The statistically significant improvement in TAD reflects a mechanical benefit that may support better implant anchorage. However, this did not translate into clinically significant differences in key outcomes such as reoperation rates or length of hospital stay, which showed no significant change. The difference in operative time was clinically modest but statistically significant. Thus, the mechanical benefit of augmentation may be clinically relevant only in select high-risk cases with unstable fractures or poor bone quality.

Compared to previous observational and biomechanical studies which showed more definitive benefits of cement augmentation in preventing implant migration and cut-out, our analysis of RCTs yielded more cautious conclusions. Studies such as those by Kammerlander et al. 10 and Salazar et al. 8 suggested improved construct stability, but their findings did not consistently demonstrate outcome improvements when subjected to rigorous meta-analysis. This highlights a gap between theoretical/mechanical advantages and measurable clinical effectiveness.

The strength of the current review lies in its adherence to the PRISMA guidelines, robust methodology, and sensitivity analysis. However, the conclusions are limited by the small number of high-quality RCTs and heterogeneity across studies. Future research should focus on stratifying patients based on fracture stability and bone quality, using standardized outcome measures such as functional mobility scores and exploring cost-effectiveness to guide targeted clinical application.

## Conclusion

Cement augmentation in internal fixation for trochanteric fractures significantly reduced the TAD, supporting improved implant positioning. However, it did not significantly reduce fixation failure, reoperation rates, or one-year mortality. Similarly, no statistically significant differences were found in terms of length of hospital stay, intraoperative blood loss, or the overall reoperation rate. Operative time was significantly longer in the augmentation group. Based on these findings, the hypothesis that cement augmentation improves clinical outcomes beyond mechanical positioning is not supported.
